# Work climate in emergency health services during COVID-19 pandemic—An international multicenter study

**DOI:** 10.3389/fpubh.2022.895506

**Published:** 2022-09-21

**Authors:** Justyna Kosydar-Bochenek, Sabina Krupa, Dorota Religa, Adriano Friganovic, Ber Oomen, Ged Williams, Kathleen M. Vollman, Maria Isabelita C. Rogado, Sandra Goldsworthy, Violeta Lopez, Elena Brioni, Wioletta Medrzycka-Dabrowska

**Affiliations:** ^1^Institute of Health Sciences, Medical College of Rzeszow University, Rzeszow, Poland; ^2^Division for Clinical Geriatrics, Department of Neurobiology, Care Sciences and Society (NVS), Karolinska Institute, Solna, Sweden; ^3^Department of Anesthesiology and Intensive Medicine, University Hospital Centre Zagreb, Zagreb, Croatia; ^4^University of Applied Health Sciences, Zagreb, Croatia; ^5^The European Specialist Nurses Organisation (ESNO), HR Arnhem, Netherlands; ^6^School of Nursing' Midwifery, Gold Coast Campus, Griffith University, Brisbane, QLD, Australia; ^7^Advancing Nursing LLC, Adjunct Faculty Michigan State University, Northville, MI, United States; ^8^Graduate School of Nursing, Arellano University, Manila, Philippines; ^9^Faculty of Education and Professional Studies, School of Nursing, Nipissing University, North Bay, ON, Canada; ^10^School of Nursing, Midwifery and Social Sciences, Central Queensland University, Melbourne, VIC, Australia; ^11^Nephrology and Dialysis Unit, San Raffaele Hospital, Milan, Italy; ^12^Department of Anaesthesiology Nursing and Intensive Care, Faculty of Health Sciences, Medical University of Gdansk, Gdansk, Poland

**Keywords:** work climate, emergency health services, COVID-19, multicenter, pandemic (COVID-19)

## Abstract

**Introduction:**

A good working climate increases the chances of adequate care. The employees of Emergency in Hospitals are particularly exposed to work-related stress. Support from management is very important in order to avoid stressful situations and conflicts that are not conducive to good work organization. The aim of the study was to assess the work climate of Emergency Health Services during COVID-19 Pandemic using the Abridged Version of the Work Climate Scale in Emergency Health Services.

**Design:**

A prospective descriptive international study was conducted.

**Methods:**

The 24-item Abridged Version of the Work Climate Scale in Emergency Health Services was used for the study. The questionnaire was posted on the internet portal of scientific societies. In the study participated 217 women (74.5%) and 74 men (25.4%). The age of the respondents ranged from 23 to 60 years (SD = 8.62). Among the re-spondents, the largest group were Emergency technicians (85.57%), followed by nurses (9.62%), doctors (2.75%) and Service assistants (2.06%). The study was conducted in 14 countries.

**Results:**

The study of the climate at work shows that countries have different priorities at work, but not all of them. By answering the research questions one by one, we can say that the average climate score at work was 33.41 min 27.0 and max 36.0 (SD = 1.52).

**Conclusion:**

The working climate depends on many factors such as interpersonal relationships, remuneration or the will to achieve the same selector. In the absence of any of the elements, a proper working climate is not possible.

## Introduction

Work climate is the quality of the work environment perceived by employees, which productivity, motivation and employee behavior affects. In the case of healthcare, it also influences the effectiveness or quality of patient care (1). The appropriate working climate is conducive to better organization of work and the proper functioning of the departments. Emergency Units employees are a very vulnerable group to the occurrence of stress. This stress can be related to both emergency situations and the need to make quick and appropriate decisions. With a good working climate, there is less risk of confusion, misunderstandings or organizational errors (1). The COVID-19 pandemic has contributed to an increase in the number of people hospitalized and situations where healthcare workers are tired, burned out, and therefore reflective and irritable. Such situations have a destructive effect on teamwork. Literature reports that a high level of care can be found in places where workers are supported by their employers (2). Considering the responsibility and constant traumatic situations faced by Emergency employees, they may develop post-traumatic stress disorder and depression. (3). A very important issue is the support that employees should receive from decision-makers, but they should also support each other and work as a team. Milton et al. Pointed to opportunities to improve attitudes among ED team members through specific organizational changes and learning from each other (4). During the COVID-19 pandemic, people were isolated from each other and only going to work often gave a sense of “normality” and the possibility of contact with other people (5). Lasalvia et al. found that one year after the COVID-19 outbreak, nurses were most likely to experience anxiety and depression, while residents were most likely to experience burnout (in terms of low work performance). Working in intensive care units was associated with an increased risk of developing severe emotional exhaustion and a cynical approach to work (6). A study by Norkiene et al. which was carried out in the UK and Lithuania, showed that psychosocial support for health professionals should be provided to prevent burnout and loss of personnel during a pandemic, as half of the study participants from two countries with different histories and health systems reported similar proportions of participants considering a career change. If these people acted as intended, it could cause serious problems with staff and healthcare in the UK and Lithuania. The threat of enormous staff shortages in such critical specialties is concerning (7). In studies by Teo et al. teamwork and a sense of appreciation at work were shown to be protective in nature and to serve as targets for the development of organizational interventions to alleviate expected underperformance among frontline health professionals. (8). The impact of a negative psychosocial work environment has been identified as a contributing factor in the occurrence of errors and adverse events in hospitals. Rasmussen et al. showed that the work environment, poor patient safety climate and increased cognitive demands were significantly correlated with adverse events (9). A key element of the literature review by Johnston was that perceptions of the work environment varied by clinical staff and study site, but the high level of autonomy and teamwork balanced the stress of high pressure and heavy workload (10). There are not many instruments to assess the work climate in health care. Most of the work is about the organizational work climate of nurses in the dimension of safety climate or ethical climate. Not much is about work climate in emergency services (1–9). This paper helps to understand the limitations of the work climate and can contribute to improving the work climate in the work of Emergency Health Services. This research allows to discover the elements which limit the work climate.

### Aim

The purpose of this study was to assess the work climate of Emergency Health Services during COVID-19 Pandemic using the Abridged Version of the Work Climate Scale in Emergency Health Services.

## Materials and methods

### Study design, setting

A prospective descriptive study was conducted in an international group. The authors followed the guidelines of the Declaration of Helsinki (World Medical Association, 2013) and STROBE (Strengthening the Reporting of Observational Studies in Epidemiology) (11).

### Participant

The study was conducted using a standardized questionnaire that was posted on the website of scientific societies. In total, the questionnaire was completed by 291 respondents from 14 countries. The authors had no influence on how many surveys would be collected and from which country. An electronic survey was available but there was no plotted number of results collected.

Two hundred and ninety-one People Working at Emergency Units from 14 Countries Participated in the Study. 217 Women (74.5%) and 74 men (25.4%) Participated in the Study. The age of the Respondents Ranged from 23 to 60 Years (SD = 8.62). Among the re-Spondents, the Largest Group Were Emergency Technicians (85.57%), Followed by Nurses (9.62%), Doctors (2.75%) and Service Assistants (2.06%). As for the Type of Employment Contract, 271 People (93.13%) Had an Indefinite Contract, 11 People (3.78%) Had a Temporary Contract (Part-Time) and 9 People (3.09%) Were on a Training Contract.

### Characteristics of the research tool

The study used the Abridged Version of the Work Climate Scale in EHS, which consists of 24 items divided into 4 elements such as: Work Satisfaction, Productivity / Achievement of aims, Interpersonal relationships and Performance at work. This scale also provided 2 open questions: What is your level of participation in the decisions of the work team? Do you have any suggestions on improving the work climate? In addition to open-ended questions, participants could rate individual statements from 1 to 5, with 1 being low and 5 being the highest possible. An abridged version of the scale consisted of 24 items. Factor 1—Work satisfaction, includes 8 items, Factor 2—productivity / achievement of aims, includes 8 items; Factor 3—Interpersonal relationships, contains 4 items, and Factor 4—Performance at work, also contains 4 items (1).

The Work Climate Scale in Emergency Services: Abridged Version, is the first short and comprehensive scale to measure four work climate factors: work satisfaction (F1), productivity / achievement of aims (F2), interpersonal relations (F3), and performance at work (F4) in the emergency services employee group. This scale is based on a 40-item work climate scale in hospital emergency services (WCSHES) (12). The original scale was developed by Lozano-Lozano (1). The study was conducted on a group of one hundred thirteen EHS workers from two different hospitals in Gibraltar Countryside County. Good psychometric properties were confirmed, obtaining satisfactory results of accuracy and reliability. Unfortunately, one of the limitations of the scale, which the authors emphasize, is its length.

The abridged 24-item version was developed by a research team led by Lozano-Lozano based on a study conducted on a group of 126 emergency health workers in Chile. It is a good tool for assessing the work climate in emergency services, also because of its brevity and simplicity. The reliability rates for the abridged version were very satisfactory (α = 0.94 and ω = 0.94) compared with α = 0.96 and ω = 0.96 for the original version. The correlation of the original version with the criteria was ρXY = 0.68, and the correlation of the abridged 24-item version with the criteria was also ρXY = 0.68, with a model fit of χ2 (248) = 367.84; *p* <0.01, RMSEA = 0.06 with an interval of 90% from 0.05 to 0.07, SRMR = 0.08, GFI = 0.9, AGFI = 0.96, CFI = 0.98, NFI = 0.95, and NNFI = 0.98 (13). So far, the scale has only been used in a validation study in Chile. Studies with this tool in other populations are recommended (1).

F1 Work satisfaction refers to feelings evoked in workers by their job and their conditions: self-confidence due to the experience, the adequacy of the workday or time for each patient, contentment with relationships with other professionals outside the group and patients and their relatives, pride, success, cohesion, or nervousness facing new circumstances (12).

F2 Productivity/achievement of aims refers to the perception of workers having everything they need to do their job or, on the contrary, lacking what they need to achieve their goals: understanding of the relevance, capabilities, and specialization of others; the value of working in a group; motivation and fulfillment of expectations; recognition of their work as a group; self-improvement; infrastructure; training and the characteristics and functioning of their service; patients' characteristics fitting with their specialization and knowledge of such characteristics; protocols; and coordination with other hospital services (12).

F3 Interpersonal relationships refer to the feelings when workers relate to other members of the group and aspects that influence such feelings: the quality of the communication, their relationship, the level of comfort, their friendship, their conflicts, being recognized for their individual contributions, having the resources they need, following a plan, participating in decisionmaking, and productivity (12).

Finally, F4 Performance at work includes everything related to the development of workers' job placement: the perceived importance of their job and capacity to decide how to improve their performance; the skills and knowledge they use; and their knowledge of their tasks and others' tasks, their individual and group limitations, and their patients (12).

The overall score is obtained by adding the score given foreach item (24 is the lowest possible score and 120 the highest). In the same way, a score can be obtained for each factor by adding up the scores for the corresponding items.

### Data collection (timing of examinations, where, consent)

The study was conducted from November 1 to December 20, 2021 as an online survey. The tool was disseminated *via* messengers, websites, and social media related to emergency medical services and emergency medicine. The tool was made available in English. The information about the study stated that the study is aimed only for people who worked in a hospital during the COVID-19 pandemic.

### Setting and procedure

By completing the questionnaire, the participants expressed their consent to participate in the study, but they could withdraw from the study at any stage of completing the form.

### Inclusion and exclusion criteria

The inclusion criteria were: health care workers from emergency units; adult; people who are working with COVID-19 patients.

### Ethical considerations

An application was submitted to the Bioethics Committee at the University of Rzeszów (KBE No. 09/05/2020) in order to obtain a positive opinion on the study.

### Statistical analysis

All statistical calculations were carried out using the IBM SPSS 23 statistical package (Armonk, NY, USA) and an Excel 2016 preadsheet. The statistical analysis included the baseline measurement adjusted to the variables, mean, standard deviation, N (%). The following rules were adopted: *p* < 0.05—statistically significant relationship. ANOVA uses F-tests to statistically test the equality of means.

## Results

### Work climate scale in emergency health services—Abridged version

Factor 1 corresponded to Work Satisfaction. The mean work climate score was 33.41 (SD = 1.52). Factor 2 was labeled Productivity / Achievement of aims and was on average 26.95 (SD = 1.11). Factor 3 corresponded to Interpersonal relationships and averaged 16.16 (SD = 1.39). The last Factor 4 was Performance at work and averaged 18.07 (SD = 1.24). In all calculations, the significance level was *p* ≤ 0.05. ANOVA uses F-tests (F) to statistically test the equality of means.

In the next stage, the work climate was measured for individual variables such as: Gender, Profession, Type of employment contract. Assuming that *p* < 0.05, no statistical significance was demonstrated in the Factors 1–4 in relation to the variables. The results are presented in Table 1.

**Table 1:** Impact of variables: gender, profession, type of employment contract on factors of work climate.

	**F1–Work Satisfaction**		**F2–Productivity/** **Achievement of aims**		**F3–Interpersonal relationships**		**F4–Performance at work**	
	**x**	**SD**	**x**	**SD**	**x**	**SD**	**x**	**SD**
**Gender**	**F = 0.63**, ***p =*** **0.42**		**F = 0.02**, ***p =*** **0.88**		**F = 0.23**, ***p =*** **0.62**		**F = 0.005**, ***p =*** **0.94**	
Female	33.45	1.50	26.95	1.10	16.14	1.36	18.07	1.25
Male	33.28	1.57	26.93	1.14	16.23	1.49	18.08	1.20
**Profession**	**F = 0.19**, ***p =*** **0.90**		**F = 0.34**, ***p =*** **0.79**		**F = 1.81**, ***p =*** **0.14**		**F = 0.35**, ***p =*** **0.78**	
Doctors	33.38	2.13	26.63	1.19	16.88	0.99	17.88	1.36
Emergency technicians	33.38	1.51	26.94	1.14	16.12	1.43	18.08	1.26
Nurses	33.61	1.59	27.07	0.90	16.50	1.04	18.18	1.09
Service assistants	33.50	1.05	27.00	0.89	15.50	0.84	17.67	1.03
**Type of employment contract**	**F = 0.92**, ***p =*** **0.39**		**F = 0.01**, ***p =*** **0.98**		**F = 0.69**, ***p =*** **0.49**		**F = 0.54**, ***p =*** **0.58**	
Indefinite contract	33.40	1.53	26.95	1.12	16.16	1.40	18.05	1.26
Temporary contract (part-time)	33.09	1.58	26.91	0.94	15.82	1.33	18.36	0.67
Training contract	34.00	1.00	27.00	1.22	16.56	1.01	18.33	1.22

P, probability value; F, ANOVA test.

Statistical significance was demonstrated in Factors 1–4 in relation to the variable which was age. Pearson correlation is between age of respondents and work climate variables: work satisfaction, productivity/achievement of aims, interpersonal relationships and performance at work. Static significance (p) values are presented in Table 2.

**Table 2:** Statistical significance of the age variable in relation to individual factors.

**Pearson correlation ( r )**	**Factors statistically significant ( p )**			
	**F1–Work Satisfaction**	**F2–Productivity/ Achievement of aims**	**F3–Interpersonal relationships**	**F4–Performance at work**
**Age**	0.03	0.04	−0.03	0.03

F1,2,3,4, factors; p, probability value.

The highest average level of work satisfaction (Factor 1) was demonstrated for workers from Canada and the lowest for workers from San Marino. For Factor 2, the highest average level was again recorded in Canada and the lowest for the residents of San Marino. In Factor 3, related to Interpersonal relationships, the highest average scores were given by employees from Netherland, while the lowest for interpersonal relationships was given by the residents of San Marino. Performance at work (Factor 4) was rated the highest by the residents of England, while the lowest was recorded by participants from Turkey. As for Factors 1–4, statistical significance was demonstrated at each level in relation to the countries that took part in the study. ANOVA uses F-tests (F) to statistically test the equality of means. The results are presented in Table 3.

**Table 3:** Correlations between factors 1–4 and the countries included in the study.

	**F1–Work Satisfaction**		**F2–Productivity/** **Achievement of aims**		**F3—Interpersonal relationships**		**F4—Performance at work**	
	**x**	**SD**	**x**	**SD**	**x**	**SD**	**x**	**SD**
**Country**	**F = 10.32, p < 0.0001**		F = 17.62, p <0.0001		F = 25.23, p <0.0001		F = 12.63, p <0.0001	
Albania	33.00	1.59	27.00	0.63	16.81	0.66	18.50	0.73
Canada	34.24	1.20	27.53	0.87	16.53	0.94	18.12	0.99
China	33.68	1.19	27.18	0.61	17.18	0.95	18.50	0.83
England	33.92	0.97	27.13	0.68	16.17	0.70	18.71	0.86
Finland	31.23	1.36	27.31	0.63	16.54	1.20	18.54	0.97
France	33.90	1.37	25.10	1.20	15.90	0.74	15.80	1.23
Germany	33.92	1.26	27.31	0.75	16.00	1.22	18.69	0.75
Italy	33.29	1.55	27.15	0.70	16.85	1.10	18.47	0.86
Japan	34.00	0.82	27.57	0.79	14.29	0.95	18.14	0.90
Netherland	33.75	0.93	27.19	0.66	17.31	0.95	18.56	1.03
Portugal	33.44	1.67	27.33	0.50	15.67	1.00	16.89	1.36
San Marino	29.29	1.50	23.00	1.29	11.57	1.27	18.14	1.35
Singapore	33.58	1.27	26.87	1.08	15.46	0.97	17.86	1.17
Turkey	33.00	0.77	27.00	0.63	16.64	0.50	15.73	0.79

F1,2,3,4, factors; SD, standard deviation; x, weighted average.

In the next stage, means and standard deviations for the entire population of people participating in the study were analyzed. In Factor 1, the respondents gave the highest scores to the statement “We strive to achieve successful outcomes,” and the lowest to “Relevance of the job of each member.” In Factor 2, the claim “Our work group is known for its productivity” scored the highest, while the claim “The merit of our good job is recognized” received the lowest score. In Factor 3, the highest rated the statement “I feel comfortable with my work group,” and the lowest “We have good communication within the group.” In Factor 4, out of 4 possible statements, the highest scores were “Our patients fit the specialty of our service” and the lowest was “I know my professional shortcomings.” The results of the mean results (x) and standard deviation (SD) are presented in Table 4.

**Table 4:** Mean (M) and standard deviation (SD) results in the overall population tested in factors 1–4.

**Factor 1**	**M**	**SD**
1. We take pride in our work	4.03	0.75
2. We strive to achieve successful outcomes	5.00	0.00
3. Relevance of the job of each member	3.24	0.72
4. We develop our skills and knowledge	4.43	0.53
5. Our work group is known for quality work	3.98	0.15
6. We have a common purpose	4.71	0.65
7. We receive the necessary training	4.09	0.31
8. The characteristics of our service are appropriate	3.91	0.48
**Factor 2**		
9. Our service works correctly	4.00	0.00
10. Our work group is known for its productivity	5.00	0.00
11. The merit of our good job is recognized	1.39	0.49
12. We are appreciated for the work we do	1.41	0.49
13. Our specialization is recognized	1.41	0.49
14. We coordinate with the other hospital services	4.92	0.34
15. We have a plan that guides our activities	3.96	0.38
16. We know what is expected in our work	4.8	0.58
**Factor 3**		
17. We have good communication within the group	3.19	0.58
18. Good relationship between members	4.22	0.57
19. I feel comfortable with my work group	4.46	0.50
20. We work in a good work group climate	4.29	0.72
**Factor 4**		
21. We understand each other's capabilities	4.44	0.50
22. I know my professional shortcomings	4.23	0.79
23. We know the functions of the members	4.46	0.53
24. Our patients fit the specialty of our service	4.93	0.29

### Testing assumptions

Despite the lack of research with the use of the original full version of the tool, it should be stated that the abbreviated version of the scale does not differ from the full version (1).

## Discussion

The study, using the Work Climate Scale in Emergency Services: Abridged Version, was divided into 4 Factors. Work satisfaction was 33.41 (SD = 1.52), productivity / Achievement of aims averaged 26.95 (SD = 1.11), Interpersonal relationships averaged 16.16 (SD = 1.39) and Performance at work averaged 18.07 (SD=1.24). The obtained factors show some similarities to those obtained in previous studies (Table 5).

**Table 5:** Standardized factors loadings obtained in the Confirmatory Factor Analysis, CFA.

**Abridged Version (24 Items)**				
**Items**	**F1**	**F2**	**F3**	**F4**
1. We take pride in our work	0.44			
2. We strive to achieve successful outcomes	0.45			
3. Relevance of the job of each member	0.48			
4. We develop our skills and knowledge	0.48			
5. Our work group is known for quality work	0.46			
6. We have a common purpose	0.40			
7. We receive the necessary training	0.46			
8. The characteristics of our service are appropriate	0.44			
9. Our service works correctly		0.45		
10. Our work group is known for its productivity		0.45		
11. The merit of our good job is recognized		0.47		
12. We are appreciated for the work we do		0.48		
13. Our specialization is recognized		0.47		
14. We coordinate with the other hospital services		0.41		
15. We have a plan that guides our activities		0.45		
16. We know what is expected in our work		0.36		
17. We have good communication within the group			0.43	
18. Good relationship between members			0.37	
19. I feel comfortable with my work group			0.46	
20. We work in a good work group climate			0.38	
21. We understand each other's capabilities				0.46
22. I know my professional shortcomings				0.48
23. We know the functions of the members				0.44
24. Our patients fit the specialty of our service				0.41

The first factor of them was responsible for job satisfaction. In a job satisfaction study, Chinese authors showed average job satisfaction, measuring it on a scale of 1–6. Among the sociodemographic variables, the occupation, education, professional status, length of service, annual income and frequency of night shifts had a significant impact on the level of job satisfaction. Stress at work, the work-family conflict, and the doctor-patient relationship also had a significant impact on job satisfaction (14). Based on the results of the study by Janicijevic et al. it can be seen that employee satisfaction, e.g., with salaries, has almost no impact on patient satisfaction (15) Linn et al. in their study they showed that hospital staff have difficulties meeting the needs of their patients if their own needs are not being met (16). Similar conclusions have also been noted by Hasenfeld et al. (17). In their own research, the respondents highly rated the Factor 1 claim that they strive to achieve successful outcomes. In the study of Javanmardnejad et. al. it has been shown that nurses working in emergency departments do not feel happy. In addition, the findings suggest that their happiness was related to their economic status and closure of duties (18).

In their own research, the respondents highly rated the Factor 1 claim that they strive to achieve successful outcomes. It follows that satisfaction is possible as long as you work on your success every day.

In a study by Joo et. al. show that Korean nurses experience a poorer security climate compared to other countries. One suggestion is to increase the satisfaction of nurses through organizational communication and to promote communication at the organizational level so that individual healthcare professionals are aware of their organisation's vision and policy (19). Similar results were obtained in the broadcasts of Poursadeqiyan et al. and Borhani et al. (20, 21).

Work satisfaction (F1) is one of the factors influencing the working environment in research conducted among the professionals of an emergency department. Work satisfaction has been shown to be related, inter alia, with burnout, ethical sensivity and leadership (22, 23).

Considering Factor two, there is little research that focuses on Productivity and Achievement of aims. In their work, Bipp et al. see a positive relationship between progress in achieving goals and commitment to work, in particular in the case of employees who have achieved goals related to the approach to results that they have planned (24). Mahmoudi et al. showed in their work that when considering achievement motivation and self-efficacy in private and university hospitals, significant differences were observed (25). Setting goals is related to self-confidence, motivation and autonomy, which is mentioned in her work (26, 27). Respondents rated the aspect of Factor 2 the highest, which was responsible for saying that they work group is known for its productivity. The problem Productivity / Achievement of aims (F2) of nurse practitioner and resident physician appeared in the McDonnell study (28).

Factor three focused on Interpersonal relationships. In the work of Orehek et al. it has been shown that interpersonal relations and the pursuit of a goal are closely related (27). Lee et al. stresses that patient safety is threatened by medical errors and adverse events related to misunderstandings among healthcare providers. In addition, the authors note that different relationships between team members lead to different collaborative behaviors, which may impact patient safety outcomes by altering team communication (29). Note the conclusions of Fernandez et al. that effective teamwork is essential to ensure safe and effective healthcare (30). Well-functioning teams adapt to rapidly changing patient factors and the environment, preventing diagnostic and therapeutic errors (31). Interpersonal relationships (F3) are considered in the dimension of communication, teamwork, leadership and decisionmaker (32–34). Our research has shown that satisfaction with the cooperation in the worhplce was rated the highest of the entire Factor 3. In the studies of Eiche et al. Satisfaction with the work of paramedics in the German EMS is low. Dissatisfaction with payment and organizational issues is widespread. The performance mindset is high, but the fear of failure is common. Current and future efforts to create an attractive working environment should reflect these findings (35).

In Factor four, Performance at work was rated. Randhawa et al. in their work, they showed a significant positive relationship between self-effectiveness and work efficiency, which suggests that the higher the self-efficacy specific for a given job, the higher the work efficiency of employees will be (31). Aboagye et al. emphasized that during the pandemic, absenteeism was to a large extent related to work efficiency. In addition, factors that interfere with performance at work must be constantly corrected including working on several jobs, which has a destructive effect on the quality and efficiency of work (36). Performance at work (F4) was described, among others, by in the works of Staempfli et al. heavy workloads, exposure to violence and conflict, and patients with a high medical condition contribute to the difficult working environment for emergency department nurses around the world. Emergency services representatives around the world report low nurse satisfaction and high levels of burnout (37).

Robinson et al. in his research, he compared the effects of productivity, efficiency and overall performance differences between lone assistants and working with residents in the emergency room. Another study found that increased productivity with decreased productivity was reported among Attendants when working with residents. Taking into account aspects related to working with residents, greater effectiveness was observed in group work than in individual work. (38). In a study by Kang et al. In the United States, emergency departments are constantly under pressure to improve operational efficiency and quality in order to obtain financial benefit and maintain a positive reputation. Depending on the structural and operational characteristics of ED, various factors can affect the relationship between performance and quality (39). Karmi et al. showed in the study that the productivity and quality of professional life of emergency nurses were at a moderate and relatively normal level, respectively. The research shows that scientific advancement does not improve the quality of professional life. It is necessary to put in place a plan to improve the productivity and quality of working life of nurses. Such plans should be implemented at the hospital level at every hospital level. The role of creating an environment suitable for a high quality of life plays a key role in the emergency department. It is necessary to conduct more developed research in this environment. (40).

## Study limitations

Through the COVID-19 pandemic, research has limitations in interacting with study participants. In addition, when assessing the pre-pandemic research, we can see the impact of the difficult period on the results of research in the EHS group. Considering the number of countries that took part in the survey and the number of people, this number is not very large, but the survey can be successfully repeated on a larger group of employees. The abridged scale is a new tool for assessing the work climate in emergency medical services. There are no studies with the use of the scale, except for the validation study, therefore in the discussion the authors referred to the research on the organizational climate conducted in professional groups with similar working conditions, mainly nurses.

## Conclusions

In response to the purpose of the research, the work climate of EHS during COVID-19 Pandemic in 14 countries was assessed using the Abridged Version of the Work Climate Scale in Emergency Health Services. The study of the climate at work shows that countries have different priorities at work, but not all of them. By answering the research questions one by one, we can say that the average climate score at work was 33.41 (SD = 1.52) at the range 24–120. It has been concluded that the emergency services staff investigated here perceive the score of the organizational climate to be low, which is represented by the low score of factors like: work satisfaction, productivity / achievement of aims, interpersonal relationships, performance at work. This raises concerns about the working conditions of emergency services staff as a low perception of work climate indicates that these conditions may not be sufficiently satisfactory to provide them with an adequate work process. This may have an impact on the quality of the health services offered. For this reason, the study of the working climate is important not only for emergency medical services personnel, but also for managers. Work climate analysis allows to verify the quality of the work environment and to determine whether and to what extent the work climate should be improved. to ensure good working conditions and good quality care. Further studies are needed to identify and clarify the specific of work climate in the emergency medical services group.

The highest average level of work satisfaction (Factor 1) was demonstrated for workers from Canada and the lowest for workers from San Marino. For Factor 2, the highest average level was again recorded in Canada and the lowest for the residents of San Marino. In Factor 3, related to Interpersonal relationships, the highest average scores were given by employees from Netherland, while the lowest for interpersonal relationships was given by the residents of San Marino. Performance at work (Factor 4) was rated the highest by the residents of England, while the lowest was recorded by participants from Turkey.

## Implications for practice

The abbreviated version of the scale reduces the time needed to complete the study, but at the same time retains its value, as mentioned in their study by Lozano-Lozano et al. (1). It is very important to conduct research on the working climate, as it can help to improve and eliminate elements that have a destructive effect on employees. Nowadays it is hard to work with the burden of a pandemic, but at the same time it should be remembered that decision-makers have a very large impact on improving the climate and quality of work in their hospitals.

## Data availability statement

The original contributions presented in the study are included in the article/supplementary material, further inquiries can be directed to the corresponding author.

## Author contributions

Conceptualization: JK-B and SK. Methodology: WM-D and SK. Software: AF and BO. Formal analysis: GW and SG. Resources: KV. Data curation: MR and VL. Writing—original draft preparation: EB and JK-B. Writing—review and editing: WM-D, SK, and JK-B. Visualization: GW, WM-D, and JK-B. Supervision: DR. Project administration: JK-B. All authors have read and agreed to the published version of the manuscript.

## Conflict of interest

Author BO was employed by European Specialist Nurses Organization. Authors KV was employed by Advancing Nursing LLC and MR was employed by Critical Care Nurses Association of the Philippines, Inc. The remaining authors declare that the research was conducted in the absence of any commercial or financial relationships that could be construed as a potential conflict of interest.

## Publisher's note

All claims expressed in this article are solely those of the authors and do not necessarily represent those of their affiliated organizations, or those of the publisher, the editors and the reviewers. Any product that may be evaluated in this article, or claim that may be made by its manufacturer, is not guaranteed or endorsed by the publisher.
